# Reasons for dropout from cardiac rehabilitation programs in women: A qualitative study

**DOI:** 10.1371/journal.pone.0200636

**Published:** 2018-07-16

**Authors:** Davinia María Resurrección, Emma Motrico, Maria Rubio-Valera, José Antonio Mora-Pardo, Patricia Moreno-Peral

**Affiliations:** 1 Department of Psychology, Universidad Loyola Andalucía, Sevilla, Spain; 2 Primary Care Prevention and Health Promotion Research Network (RedIAPP), Malaga, Spain; 3 Research and Development Unit, Institut de Recerca Sant Joan de Déu, Barcelona, Spain; 4 Centro de Investigación Biomédica en Red Epidemiología y Salud Pública (CIBERESP), Barcelona, Spain; 5 Hospital Nuestra Señora de Valme, Sevilla, Spain; 6 Instituto de Investigación Biomédica de Málaga (IBIMA), Malaga, Spain; Toronto Rehabilitation Institute, CANADA

## Abstract

**Background:**

Empirical evidence has shown that cardiac rehabilitation programs are effective in reducing morbidity and mortality, improving quality of life in patients with cardiovascular disease. Despite the benefits, women have a high cardiac rehabilitation dropout rate. Our aim was to explore women’s perceptions about the reasons they faced for dropout from these programs.

**Methods:**

Semi-structured interviews were conducted with women (*n* = 10) after dropping out from three different cardiac rehabilitation centers in Spain. In addition, a focus group and a semi-structured interview with cardiovascular professionals were conducted. From a grounded theory perspective, thematic analysis was used to derive themes from interview transcripts.

**Results:**

The women were between 41 and 70 years. We identified five general themes that illustrated reasons for cardiac rehabilitation dropout: intrapersonal reasons (self-reported health, self-reported mental health, health beliefs); interpersonal reasons (family caregiver role, work conflicts); logistical reasons (transport, distance); cardiac rehabilitation program characteristics (perception of the objective of cardiac rehabilitation, exercise component, inconvenient timing, cardiac rehabilitation equipment); and health system reasons (financial assistance for transport, long waiting list). The cardiovascular professionals found barriers to cardiac rehabilitation completion similar to those found by the women.

**Conclusions:**

In order to prevent cardiac rehabilitation dropout in women, modular and flexible programs are needed. In addition, the inclusion of primary care centers or community resources could improve cardiac rehabilitation completion in women. Psychological assessment and counseling during cardiac rehabilitation should be included as an essential part of the programs and recommended for those women with depressive symptoms. Finally, improved financial assistance for transport from the health system is essential.

## Introduction

Cardiovascular disease (CVD) is the leading cause of death in women (51%) [1]. Moreover, CVD presents a 5% prevalence in women, increasing with age, reaching 18.8% among women >80 years [1,2]. Due the high recurrence of a reinfarction within the next five years (22%), secondary prevention of a new cardiac event is essential [3]. Cardiac rehabilitation (CR) programs are multifaceted interventions designed to optimize clinical outcomes in cardiac patients, focused on exercise, medication management, nutritional counseling, risk factor management, and psychosocial counseling [4]. CR is considered a Class IA recommendation in clinical practice guidelines for women [5,6]. Empirical evidence has shown that it is effective in reducing morbidity and mortality, improving quality of life in patients with CVD [7,8]. However, CR adherence is below the recommended levels [9,10]. Specifically in Europe, the EUROASPIRE IV Survey showed that only one-third of patients have access to CR in Europe [11]. Enrollment in CR does not guarantee completion of the program. A recent narrative review found that in high income countries CR dropout rates ranged from 12–56% [12]. In Spain, less than 7% of eligible patients participated in CR and participation rates for women are lower than recommended in clinical guidelines [13,14].

Two reviews found that CR completion was associated with individual and contextual factors such as the perceptions of CR programs, being male, younger than 65 and having transport [15,16]. Specifically in women, recent systematic reviews found that CR dropout is influenced by multilevel barriers such as intrapersonal barriers, interpersonal barriers, logistical barriers, health system barriers, or CR program barriers [17–19]. However, the only qualitative studies included in these reviews were related to experiences or reasons for women’s participation into CR [20–22], and there were no specific qualitative studies focused on women’s reasons for dropping out from CR. In this context, qualitative studies are useful, eliciting needs of patients and providing new perspectives in health care [23]. Therefore, the objective of this study was to understand the reasons for CR dropout from the perspective of women who dropped out from the program.

## Materials and methods

The study was approved by the Andalusian Biomedical Research Ethics Portal. All the participants were informed that they could withdraw from the study at any time without providing a reason. All the participants were informed about the purpose of the study, and provided oral and written informed consent. All data collected were treated confidentially and anonymized. The reporting of data in this study follows the consolidated criteria for reporting qualitative research (COREQ) checklist [23].

### Study design and sample

This study was carried out from a grounded theory perspective. Due to the lack of studies to date evaluating reasons for CR dropout in women, we ascertained first-person opinions of women who have had this experience.

The first author (DMR), female, aged 29 with previous experience in clinical psychology, conducted semi-structured interviews. The second author (EM), white female, aged 41, was the group facilitator in the focus group, with previous research experience in conducting focus groups; and was observed by the first author.

For semi-structured interviews, we employed an intentional and theoretical sampling to recruit the participants. Following maximum variation sampling, the inclusion criteria for the study were: (1) women with a principal diagnosis of CVD; (2) having a referral to a CR program; (3) being able to speak and understand Spanish; (4) no cognitive impairment; and (5) CR dropout at any point in the program. Gifts as compensation were offered to the participants at the end of the interview. Nurses working in three CR centers in Andalusia (Spain) conducted the recruitment of participants: Nuestra Señora Virgen de Valme Hospital (Seville), San Cecilio Hospital (Granada), and Puerta del Mar Hospital (Cadiz). Then, the first author contacted the potential participants by phone and provided further information about the study. If the individual was interested in participating, an appointment for the interview was scheduled. We continued to sample, conduct interviews, and analyze data until no new information was obtained and the themes in subsequent interviews achieved thematic saturation. Between November 2015 and December 2016, 12 women were invited to participate. One of them refused due lack of interest in the study, and we could not contact the other woman because she moved out of the country. Due to low rates of women referred to CR, the process of recruiting participants took several months.

With the objective of data triangulation, a cardiovascular professional focus group and a semi-structured interview were carried out. Employing intentional sampling, we recruited cardiologists, nurses, and physiotherapists from the CR centers where women have participated. First, we contacted a cardiologist who proposed several professionals from the CR field. Then, the first author contacted them by email and scheduled a meeting suitable for most of the participants. Of the nine professionals invited to participate, three could not participate due to schedule issues. One of the professionals who could not participate in the focus group participated in a semi-structured interview. From the cardiac professionals that participated in the focus group, one cardiologist was in charge of referring patients and the other cardiologist was at her first experiences with CR programs. Three nurses were case managers in the programs and the rest of professionals participated daily in the CR program activities.

### Data collection

Face-to-face semi-structured interviews were conducted in participants’ homes or coffee shops. We collected sociodemographic data using a self-administered questionnaire at the end of the interview. We used a semi-structured interview guide that included open-ended questions to learn about the participants’ views of CVD and CR experiences. Questions were focused on the participants’ views of the reasons they had to dropout from CR (interview guide in Table 1). The main topics supported the interview as overarching questions to start the dialogue. The interviewer used reflective probes to encourage participants to clarify and expand on their statements. An iterative process was followed during the course of the study, and the interview guide was modified with the content of previous interviews.

**Table 1:** Semi-structured interview guide.

a. Health beliefs (causes, consequences, treatment) What happened after you suffered the cardiovascular event? What do you think could happen if you do not finish the cardiac rehabilitation program?
b. Treatment health beliefs (benefits/barriers). Previous treatment experiences (your own, the environment) How was your experience in the cardiac rehabilitation program? If you have had experience with other rehabilitation programs, how has that experience been?
c. Cardiovascular professional-patient relationship When you were still in the hospital, did anyone tell you about the cardiac rehabilitation program? Who talked to you about the program?
d. Personal and psychosocial factors In your case, what things/situations influenced you to dropout of the cardiac rehabilitation program?
e. Practical factors Where is the cardiac rehabilitation program located? What implications does this have for the program? Did it have any influence that there were other women with you in the program?

Professionals were invited to Universidad Loyola Andalucía to participate in the focus group, and transport costs to the center were reimbursed. The group facilitator used a semi-structured interview guide for the focus group (Table 2). The main discussion was focused on barriers and facilitators that women faced for dropout from CR programs. We collected sociodemographic data at the end of the focus group. The professional semi-structured interview followed a guide similar to the one employed in the focus group.

**Table 2:** Focus group interview guide.

Based on your opinion as health professionals, what are the barriers associated with the low participation of women in cardiac rehabilitation programs?
What initiatives or actions could be implemented to increase participation in cardiac rehabilitation programs?

### Data analysis

The interview guide was tested in a pilot interview. No significant changes were made and the pilot interview was included in the analysis. All interviews and the focus group were digitally audio-recorded and transcribed verbatim by the first author (DMR). The semi-structured interviews and the focus group were then analyzed independently by two investigators (DMR, MRV, or PMP) in accordance with grounded theory and, then, triangulated. Data was analyzed manually by coding it. First, researchers read all transcripts and identified meaningful segments, selecting into different subthemes. According to the grounded theory perspective, themes and subthemes were inductively generated and grouped by their thematic similarity. Second, the investigators grouped each subtheme into general themes. General themes were discussed among the entire team of investigators. Finally, representative quotes were collectively selected following domain 3 from COREQ checklist [23].

To ensure the rigor of the results obtained from the semi-structured interviews and the focus group, after data analysis, the participants were contacted and asked to verify the accuracy of the researchers’ understanding of their main points, views, and beliefs. Three participants responded. Two of them agreed with the data obtained, and one gave some clarifications that were included in the data analysis.

## Results

### Characteristics of the cardiac rehabilitation programs

Women dropped out from three different hospital-based CR programs. Programs took place during mornings. Number of sessions ranged from 10 to 36 sessions, based on the patient’s risk stratification. Exercise classes were twice or three times per week. Educational classes ranged from three to eight. Health professionals involved were cardiologists, primary care physicians, nurses, and physiotherapists, and one of the programs included a voluntary psychologist. Cardiologists were those in charge of referred the patient to the programs, and the main nurse of each program was the responsible for contacting patients.

### Characteristics of the participants and professionals

Ten semi-structured interviews were carried out with the women. The participants were between 41 and 70 years old and all were white. Seven participants were married or living with a partner and three were divorced; seven had primary studies and three had secondary studies. Seven were working as housewives, two had paid work, and one was unemployed; eight reported having comorbidities such depressive symptoms, diabetes, arthrosis, or hypertension. The period from the cardiac event prior to start the program was from a minimum of a month to a maximum of seven months. With respect to the dropout moment, two women dropped out after the first session, three after the initial sessions, and other three did not complete by the end.

Seven cardiovascular professionals agreed to participate in the study. The professionals were between 28 and 64 years of age and all were white. There were four women; four nurses, two cardiologists, and a physiotherapist. Three had less than ten years of experience in the cardiovascular field.

Semi-structured interviews with the women lasted from 22 to 72 minutes, with a median duration of 42 minutes. The focus group lasted 124 minutes, and the professional semi-structured interview lasted 35 minutes. We organized information into themes and subthemes to understand barriers for CR completion. The results of the semi-structured interviews are presented in five main themes and 13 subthemes (see Table 3).

**Table 3:** Semi-structured interview themes and subthemes that addressed reasons for CR dropout reported by women.

General themes	Subthemes
Intrapersonal reasons	Self-reported health
	Self-reported mental health
	Health beliefs
Interpersonal reasons	Family caregiver role
	Work conflicts
Logistical reasons	Transport
	Distance
CR program characteristics	Perception of the objective of CR programs
	Exercise component
	Inconvenient timing
	CR equipment
Health system reasons	Financial assistance for transport
	Long waiting list

### Results of the qualitative analysis from the semi-structured participant interviews

#### Intrapersonal reasons

Self-reported health: Women underscored that not feeling recovered from the cardiac event, feeling tired or feeling chest pain during CR exercise were reasons for CR dropout.

I told him that I wasn’t well enough yet to start cardiac rehabilitation, because I got tired a lot and then I tried using some of the machines there in rehabilitation, impossible. I knew that I couldn’t do it. Then I said, look, let’s leave it, let’s leave some time.

– *70 years old*.

Self-reported mental health: Women reported lack of motivation to participate in CR, feeling depressed, anhedonia, or affliction after suffering CVD.

I don’t know, I broke down in a way that I did not … I did not feel like … I don’t know. I wasn’t motivated, I don’t know, I wasn’t, I wasn’t well. I didn’t feel well.

– *48 years old*.

And now seeing you like this, psychologically it affects me. A lot. A lot. Look I’m in good spirits and all that, but it affects me. I’ve been a little down, I do not feel I’m even 70% of what I was. All that, psychologically, it’s that it also has an impact.

– *70 years old*.

Health beliefs: Beliefs that the cardiovascular condition was not severe and attributing the cardiovascular event to external causes were reported as reasons for dropping out from CR.

But you see, I don’t even know what to say. If they’d said, look at me I have a bypass, they gave me … mmm, I have a STENT, I have something in my heart, well, look, I would have thought twice about it. But I’m fine. If it was from the birth control pills. Mmm, I do not need …

– *47 years old*.

#### Interpersonal reasons

Family caregiver role: Women reported that their caregiver role interfered with their participation in CR, having to dropout from the program.

Man, not me, I may miss a week … but I can’t really, because my family depends on me.

– *51 years old*.

It’s that, first it’s being a caregiver and then it’s me.

– *50 years old*.

Well … they put me in the mid-morning one, I think it was the one they put me in. But of course, then that was not good because I had to … to be here for the meal and all of the children.

– *60 years old*.

Work conflicts: Work commitments were cited by women as reasons for CR dropout. Finding a job during the CR period or the impossibility of leaving the job were reasons for dropping out from the program.

I could not finish it all because I got a month of work. And I had to take advantage, I had no choice. Because my husband is unemployed, so I had to take advantage of it. I had to leave.

– *47 years old*.

The truth is I was very happy, but impossible. I can’t go. I feel very bad, I can’t. I can’t go anywhere, now in December I have to go to the cardiologist to the six-month visit already. So now I have to find someone, let’s see who I leave …there.

- *51 years old*.

#### Logistical reasons

Transport: Women reported transportation barriers for attending CR programs. The need for public transport, the timing of transport, or the unavailability of access to a shuttle service to and from the program was cited as reasons for dropping out from CR.

Also from the buses, what time do I get here? At 4 or 5 in the afternoon?

– *60 years old*.

Also problems with the commute.

– *60 years old*.

But then from here to catch the buses, and that’s a lot of inconvenience.

- *60 years old*.

Distance: The geographical distance of the CR program was cited as a reason for CR dropout. Women reported that they would not dropout from the program if it was located in their town.

If it had been here in the village then no … there wouldn’t have been any problem.

– *60 years old*.

Here in PR apparently there isn’t, nor in the university hospital either because from there I was sent to C.

– *47 years old*.

#### Cardiac rehabilitation program characteristics

Perception of the objective of CR programs: Some women reported being uncertain about the purpose of CR and having misconceptions about the program.

For me it wasn’t positive. For me it wasn’t positive. I would have liked … I expected … something else from that.

– *63 years old*.

He told me a rehabilitation course. I wanted rehabilitation. I didn’t want information.

– *63 years old*.

I don’t know how long the course was, I don’t remember, but I didn’t finish it. But that was why, because I didn’t … I didn’t, didn’t see any benefit.

– *63 years old*.

Exercise component: Expecting more exercise and less counseling were reported as reasons for CR dropout. In this line, women reported that they found CR too boring and with too many educational sessions.

I wanted to, because I liked the gym more, but I went there the … I like the treadmill. And a lot of talk, lots of talk, but very repetitive, because okay when the talks, are enjoyable, but when he repeats, repeats, repeats.

– *63 years old*.

What I wanted was sports, because what I wanted to do was strengthen my heart.

– *63 years old*.

Inconvenient timing: Several women reported that the timing of the CR was the reason for dropping out from CR. Moreover, women reported preference for going to CR during the afternoon and not in the mornings.

Which is what I don’t agree with, the schedule.

– *47 years old*.

I would have … Yes. In the afternoon I have more … Flexibility.

– *51 years old*.

CR equipment: Women reported that the need for more equipment in the CR program was a reason for dropping out from CR. Moreover, the participants pointed out the need for better-adjusted equipment.

But those high bikes with the back … no, I wasn’t comfortable. And … when, of course, I couldn’t be, I had to be a quarter of an hour on the bicycle and a quarter of an hour on the treadmill.

– *63 years old*.

To take turns, because there weren’t enough for everyone. Well, after I did the treadmill I would go home.

– *63 years old*

"E: So, do you think that if there had been other types of machines?"

Yes, yes. I would have stayed. I would have.

– *63 years old*.

#### Health system reasons

Financial assistance for transportation: Cost of public transport and travel cost were reasons reported for CR dropout for women who were in impaired economic circumstances. In addition, women reported that financial assistance for transport was insufficient, and they had to dropout from CR because of this.

It’s not 5 euros a week, nor 5 euros every 15 days, it is 5 euros every time I went and if you go three times a week, it’s 15 euros a week. And then I went to the first, the first few times but I haven’t gone any more. Not because I haven’t wanted to but because I have no means to go.

– *47 years old*.

Then, then told us they would pay us. But of course, then … uf, who knows when they were going to pay you.

– *48 years old*.

They, when you finish the rehabilitation, you request with the bus tickets, you request, but they say that it takes … that takes a long time.

– *47 years old*.

Long waiting list: A woman informed that the delay between the cardiac event and the referral to CR was a reason for dropping out from the program.

But of course, what happens is that in that month … I thought it was going to be in that month when I was sort of on vacation. Then I could have gone and been happy to because the children, I say "listen up …" and my husband would have watched them. A case like this, right? But of course since they’ve taken about three months to call me because there are a lot of people … well …

– *51 years old*.

### Results of the qualitative analysis from the cardiovascular professional focus group and semi-structured interviews

The professionals highlighted that there might be mistaken beliefs about the negative effects and consequences of CVD in women. These beliefs are widespread among professionals, women patients and their social and family environment, leading to lesser importance being placed on participation in CR programs.

In women there is a perception that the undesirable effects and sequelae of ischemic heart disease are lower than in men (…). Therefore, less emphasis is placed on the need to participate in prevention and cardiac rehabilitation programs.

- P5

The cardiovascular professionals pointed out that women with cardiac events are usually older, have pain, have comorbidities, and are physically impaired. These characteristics might influence the exercise component. Moreover, professionals reported that women usually present more problems doing the exercises due to not having exercised previously.

And when you’re going to do the … program with her, on a physical level these are women who are very deteriorated. That is, they are women who have two good days and four bad days. Two good days, four bad days. There is no upward linear progression where you say ah that’s good, come on!

–P4

And anyway there’s one thing women have a hard time with. Physical exercise. Above all, in people already of a certain age. Let’s see, they haven’t done any physical exercise in their life, okay? So, I’m going to go do physical exercise?

–P2

Regarding the women’s family caregiver role, professionals highlighted that women try to maintain their role in the family and may find themselves unprotected by their family network.

A woman has a heart attack, and there are only very few men who are attentive to her.

–P4

Vacation is unthinkable. Me on vacation mmm, they can’t come, especially the grandmothers, and what do I do with my grandchildren?

–P2

The professionals suggested that women dislike being alone in a predominantly male CR group. They reported that they usually incorporate at least two women in a CR group, trying to prevent women from dropping out of the program. However, none of the women reported being the only woman as a reason for CR dropout, even when the interviewer explored this.

I never put, well because maybe I have no choice, but … I try never to put a woman alone in a group of men. Provided there is another woman.

–P2

With respect to financial aid for transportation, the professionals pointed to the cost of public transport and the delay from the administration for covering the costs as barriers to women completing CR.

Usually the problem is where you are coming from, that you have certain expenses. The expenses are transfers, even if they are within the same capital you have to take a bus, although we tell them that they can walk. But that is an expense, if they come from a village, especially from a village, this increases the expense a lot. Although the public health system pays a little, but … it pays part, mmm, you get paid afterwards.

–P7

## Discussion

### Main results

To the best of our knowledge, this is the first study to describe the women’s perspective of their reasons for CR dropout. In addition, there was no previous qualitative study focused on this topic within the European context. A qualitative approach was used to acquire better understanding of the complex interplay of barriers previously described in the literature. All these reasons can be framed in a 5-level ecological model: a) intrapersonal; b) interpersonal; c) logistical; d) CR program characteristics; and e) health system reasons. Similar to previous reviews [16–19] women reported health problems, transport issues and the setting and timing of the program as reasons for dropout from CR programs. These reasons interact and might influence each other. In order to prevent CR program dropout in women, multilevel health policy solutions must be addressed.

As described in previous systematic reviews [16–19] women described self-reported mental health reasons for CR dropout. Following the recommendations of depression screening in preventive guidelines for cardiovascular disease in women [24], all CR programs should incorporate psychological assessment and specific counseling. In addition, giving more information about this component during first sessions, may improve the awareness of psychological barriers in women.

Offering different CR settings could decrease dropout rates. As a recent overview found, community-based or home-based CR programs are equally effective with similar health costs as traditional CR [19,25]. In this line, we found that CR programs reported in this study took place in the mornings. Participation in CR conflicts with familial demands and the women’s family caregiver role. These reasons seem to interact with the inconvenient timing of CR programs, at the time when women usually do housekeeping or are in the care of children or grandchildren. In order to prevent CR dropout, programs CR settings in primary care centers could improve adherence, by offering more flexibility for participation, and providing different schedules during mornings and afternoons with adaptive modular components.

Transport and distance reasons for CR dropout have been widely reported in literature [15–19]. These barriers could be addressed with the use of flexible CR formats or with primary care CR programs. Related to this, health system policies should incorporate better financial aid for those women with low socioeconomic status, such as better access to a shuttle service to and from the CR program, to defray the expense of transport.

Triangulation of data from women and cardiovascular professionals has highlighted that professionals might not be aware of some of the barriers that women face for CR completion. Cardiovascular professionals have the perspective that women encounter more difficulty with the exercise component, which may lead them to not appropriately encourage those women with higher exercise capacity. However, women reported the need of more exercise and less counseling. One of the practical suggestions that could be implemented to suit specific women’s needs is that exercise and counseling components should be modular. Moreover, professionals should consider offering alternative exercise formats, such as exercise in the community, with the objective of incorporating these exercises into the women’s lifestyle. Finally, women need to perceive more accurately the value and need for completing CR. Nursing education about CR and specific assessment of functional status are essential components that may promote CR completion [26].

### Limitations

As study limitations, we should point out that the study was carried out in one Spanish region and, consequently, some of the vocabulary may have a strong cultural component. A further possible limitation could be that our sample included only Andalusian women who had dropped out from CR. Despite that, reasons reported by women are similar of those that have appeared in the literature, allowing to presume similar results in other countries [6]. The study did not include immigrant women, limiting the ability to comment on reasons for dropout for these participants. Despite these limitations, to be able to offer women the best CR it is vital to understand their perceptions of the reasons they faced for dropout from these programs. Future research should address reasons for dropout from CR in other populations. For example, qualitative studies could explore the adherence process in immigrant populations. In addition, future studies could identify reasons for nonparticipation in CR programs from the perspective of women.

## Conclusions

This qualitative study of women’s perceptions of their reasons for dropping out from CR suggests that CR programs need to be flexible and modular in order to promote completion by female participants. The study contributes to the knowledge identifying some of the reasons women set forth, including their preferences for more flexible program schedules, and their expectation of more exercise. Psychological assessment and counseling during CR are also essential for those women who need it. Health system policies should include improved financial assistance. Finally, CR program planning strategies should incorporate communities and primary care centers.
